# Using formative research to develop MNCH programme in urban slums in Bangladesh: experiences from *MANOSHI*, BRAC

**DOI:** 10.1186/1471-2458-10-663

**Published:** 2010-11-02

**Authors:** Syed Masud Ahmed, Awlad Hossain, Marufa Aziz Khan, Malay Kanti Mridha, Ashraful Alam, Nuzhat Choudhury, Tamanna Sharmin, Kaosar Afsana, Abbas Bhuiya

**Affiliations:** 1 Research and Evaluation Division, BRAC 75 Mohakhali, Dhaka 1212, Bangladesh; 2 Public Health Sciences Division, ICDDR, B 68 Shaheed Tajuddin Ahmed Sharani, Dhaka 1212, Bangladesh; 3 BRAC Health Programme, BRAC 75 Mohakhali, Dhaka 1212, Bangladesh

## Abstract

**Background:**

*MANOSHI*, an integrated community-based package of essential Maternal, Neonatal and Child Health (MNCH) services is being implemented by BRAC in the urban slums of Bangladesh since 2007. The objective of the formative research done during the inception phase was to understand the context and existing resources available in the slums, to reduce uncertainty about anticipated effects, and develop and refine the intervention components.

**Methods:**

Data were collected during Jan-Sept 2007 in one of the earliest sites of programme intervention in the Dhaka metropolitan area. A conceptual framework guided data collection at different stages. Besides exploring slum characteristics, studies were done to map existing MNCH service providing facilities and providers, explore existing MNCH-related practices, and make an inventory of community networks/groups with a stake in MNCH service provision. Also, initial perception and expectations regarding the community delivery centres launched by the programme was explored. Transect walk, observation, pile sorting, informal and focus group discussions, in-depth interviews, case studies, network analysis and small quantitative surveys were done to collect data.

**Results:**

Findings reveal that though there are various MNCH services and providers available in the slums, their capacity to provide rational and quality services is questionable. Community has superficial knowledge of MNCH care and services, but this is inadequate to facilitate the optimal survival of mothers and neonates. Due to economic hardships, the slum community mainly relies on cheap informal sector for health care. Cultural beliefs and practices also reinforce this behaviour including home delivery without skilled assistance. Men and women differed in their perception of pregnancy and delivery: men were more concerned with expenses while women expressed fear of the whole process, including delivering at hospitals. People expected 'one-stop' MNCH services from the community delivery centres by skilled personnel. Social support network for health was poor compared to other networks. Referral linkages to higher facilities were inadequate, fragmentary, and disorganised.

**Conclusions:**

Findings from formative research reduced contextual uncertainty about existing MNCH resources and care in the slum. It informed *MANOSHI *to build up an intervention which is relevant and responsive to the felt needs of the slum population.

## Background

Despite impressive gains in the health sector since independence in 1971, Bangladesh remains a country with high maternal, neonatal and infant mortality (322, 45, and 52 per 100,000 live births respectively) compared to many low-income countries with similar conditions [1,2]. Around 60% of the pregnant women receive mostly a single antenatal care visit from a medically trained provider while only 18% of childbirths are attended by skilled providers. In urban areas, the condition in the slums is worse than the non-slum areas with respect to ante-natal care (ANC) visit to a medically trained provider (62% vs. 85%), delivery at a facility (12% vs. 46%), and skilled assistance at delivery (18% vs. 56%) [3].

*MANOSHI *(acronym for mother, neonate and child in Bangla) is being implemented by an indigenous non-government organization (NGO), BRAC (http://www/brac.net), since 2007 and funded by Gates Foundation for five years [4]. It aims to reduce the morbidity and mortality of the mothers, newborns, and children in urban slums of Bangladesh through development and delivery of an integrated, community-based package of essential health services. In the short run, the programme seeks to create demand for services in the community through increasing knowledge, building capacity of the service providers (including capacity for home-based delivery and neonatal care), and develop effective referral linkages for emergency obstetric care (EmOC). To sustain these activities, the programme in the medium-term seeks to strengthen and sustain referral linkages with local health facilities in public and/or private sectors, involve and strengthen all stakeholders for effective participation, and develop a support network in the community to facilitate and sustain post-grant activities. These activities with different time perspectives are undertaken within the broader framework of the three "**D**s": **D**elay in decision-making to seek emergency obstetric service, **D**elay in transportation, and **D**elay in receiving services at place of referral [5].

Formative research is used to extract information on beliefs, values, attitudes, knowledge and behaviours of target audience related to their health problem, and seeks to answer questions about the context that influences, and is influenced by, these individual factors [6]. It uses both qualitative and quantitative methods for information gathering which is limited in time (brief) and resources (small). As such, the methods employed are 'usually less definitive than the methods a study would employ ... to test hypotheses, establish external validity...' [7]. This is plausible because the 'aim is not to answer questions about intervention definitively, rather to decrease uncertainty about what the intervention should be'. Thus, during the inception phase of *MANOSHI*, formative research was done to understand the context of programme intervention in the slums (e.g., socio-demographic characteristics of the slum, key influential people for health related matters, existing health networks/resources and services, current MNCH practices and perceptions etc.), develop and fine-tune the intervention components to reduce uncertainty about anticipated effects (e.g., community acceptability of the programme initiated delivery centers or "birthing huts"), and help researchers familiarize with the ground realities and challenges of doing research in the slums.

### Conceptual framework

Different models are proposed for formative research as it provides direction to the model. These include social cognitive model [8], social marketing model [9], and the ecological model [10]. According to the first model, people learn by seeing others with influences from the environment, behaviour, and cognition--- all interdependent factors. Social marketing model emphasizes 'audience segmentation, channel identification, and development of appropriate message.' The most useful model in the health sector has been the Ecological model [6] which focus information gathering at specific levels such as individual, interpersonal, organizational, community and policy levels that may be amenable to change by specific interventions.

For *MANOSHI*, we have adopted the ecological model with some modifications. We wanted to keep the studies focused on the state of MNCH care in urban slums. Thus, instead of the five levels described above, we collected data at three specific levels, such as,

•Community level (e.g., community characteristics, community groups/networks and its potentials for supporting MNCH programmes)

•Organisational level (e.g., existing providers and facilities providing MNCH care, and referral linkages)

•Individual level (e.g., sociodemographic characteristics and existing knowledge and practices, perceptions of pregnancy, delivery, and early "birthing huts")

This paper describes how information gathered from different streams of formative research was used to understand the context and existing resources, and thereby develop and refine *MANOSHI *intervention.

## Methods

The formative research for the *MANOSHI *programme was mainly done in one of the earliest sites of programme intervention---the *Korail *slum under Gulshan *thana *of Dhaka City Corporation during Jan-Sep 2007, in several phases. Both qualitative and quantitative methods were used to collect data. The study group was composed of researchers from both BRAC and ICDDR,B (http://www.icddrb.org) and was led by two principal investigators. Where needed, social science graduates were recruited and trained to help data collection under close supervision of the respective lead researchers.

We adopted a participatory approach in implementing the formative research process. It started with transect walks of the *Korail *slum along several routes to have an understanding about its geographic and socioeconomic characteristics, household clustering, etc. During transect walk, informal discussion with the slum dwellers helped identify key informants. Later, a participatory rural appraisal (PRA) was conducted with these key informants and slum-dwellers from different corners of the slum to prepare a health resource map identifying the location of the healthcare providers and facilities. This map was used in the listing and mapping exercise to identify all types of practitioners (both in formal and informal sectors) who are active in the slum. A sample of different types of providers was drawn purposively from the above list and in-depth interviews and focus group discussions (FGDs) were conducted with them to prepare an inventory of available MNCH services in the slum. Also, a listing exercise was done to identify the key persons to whom the slum dwellers go for advice and financial help when a health emergency arises. This was supplemented by information on different existing social networks including health networks.

In another stream, free listing and pile-sorting was done to probe people's perceptions of normal pregnancy and labour, and in-depth interviews with pregnant and lactating women were done to elicit current MNCH practices in the community. Finally, participant observation was done to collect data on newly launched *MANOSHI *delivery centres (nick named 'birhting huts') to see how these were embedded and accepted in the community. A number of mini-surveys were done in a number of the above studies to supplement qualitative information e.g., audit of health facilities, sociodemographic characteristics of the respondents, etc.

Triangulation of data from different sources was achieved by eliciting information on a particular issue from different sources e.g., data on current MNCH practices were collected primarily from the pregnant and lactating women, but during interviews with the healthcare providers (for inventory of available MNCH services study) and the community people (for health network study), the respondents were also asked about prevailing MNCH practices in the community. A brief summary of the different methods with its main focus, respondents, data collected, and the output/analysis is given in Table 1.

**Table 1:** Summary of methods used in different studies in the *Korail *slum under formative research for *MANOSHI*

Methods	Major focus	Sample/respondents	Data collected	Analysis/output
Transect walk (along different routes) and informal discussion	To explore slum characteristics	Slum dwellers met during transect walks	Physical, socioeconomic and other characteristics; knowledgeable people in the community	Describe slum characteristics; key informants identified

Participatory Rural Appraisal (PRA) (1)	To identify location of the MNCHcare providers and facilities	Key informants; slum dwellers from different corners of the slum	Location of the healthcare providers/health facilities	Health resource map

Listing and mapping exercises using health resource map (67)	To identify all MNCH health care providers	Key informants; snow-ball sampling to identify more key informants	Data on currently practicing healthcare providers	A list of all healthcare providers including those working in the facilities

In-depth interviews (17) and FGDs (5) of health care providers from list	To elicit data on available MNCH services in the slum	Purposive sample of different types of healthcare providers	Services offered, patient flow, criteria for and process of referrals etc.	An inventory of MNCH services available in the slum

Listing exercises (17) and social network analysis (3)	To identify influential persons in the slum for health-related needs/existing social networks	Key-informants/purposive sample of slum dwellers	People with political and other affiliations/social networks for health, education, economic etc.	Key influential people identified/network analysis with UCINET software

Free listing (60) and pile-sorting (20); [+in-depth interviews (14)]	To elicit perceptions of slum community about pregnancy and delivery	Purposively selected sample of women and men	Perception of normal pregnancy and delivery, fears associated with delivery, etc.	Similarities and dissimilarities between women and men's perceptions and priorities

In-depth interviews with women (16)	To elicit existing MNCH practices	pregnant women and lactating women selected purposively	Practices and rituals during pregnancy, delivery and post-partum	Inventory of existing MNCH practices

Observation	How 'birthing huts' are embedded in the slum community	'Birthing huts' in four different slums including *Korail*	Physical facilities; awareness, perceptions and acceptability of 'birthing hut' services;	Content analysis of observational data

NB. A number of mini-surveys were conducted in a number of the above studies above e.g., audit of health facilities, socio-demographic characteristics of the respondents etc. which is not shown separately in the table

### Thematic areas of study

Data in different studies were gathered at three different levels (community, organizational and individual) as described in the conceptual framework. These were then organized into four key thematic areas according to programme's need to develop and fine-tune intervention in the inception phase. These were: i) community profile, influential people/social networks relevant to MNCH care; ii) currently active MNCH service providers/facilities; iii) perception of pregnancy and delivery, existing MNCH practices; and iv) 'birthing huts' (*MANOSHI *delivery centres).

### Ethical approval

The individual studies passed through the institutional review process at the Research and Evaluation Division, BRAC (by Internal Review and Publication Committee, IRPC) for ethical clearance. No invasive technique was used in these studies. Data were collected after obtaining informed verbal consent from the respondents who were not comfortable about signing any document. The written consent form was read out and explained to the respondents and when the investigator was satisfied that the respondent understood it including its implications, and agreed to participate, only then s/he was included in the respective study. Anonymity of the respondents was maintained at all stages of data entry and analysis.

## Results

A review of the results from different streams of studies are presented according to the key themes identified above. The details can be found in the respective individual studies.

### Community characteristics: the Korail slum

The *Korail *slum is one of the biggest slums in metropolitan Dhaka with a population of about 100,000 spread over 586 acre of government land [11]. It is shaped like a peninsula, surrounded by lakes on three sides. The early inhabitants came mainly from the nearby Comilla district, followed by people from other districts. The main reasons behind migration into the slum were poverty arising out of river erosion, flood, lack of land and employment opportunities, etc. People coming from different districts tend to live together in clusters in an attempt to preserve their dialect and culture. However, this results in poor development of social cohesion and support network. The slum population is highly unstable, in- and out-migration in the slums is a continuous phenomenon, and the constant threat of eviction is part of slum life.

The slum has no legal electricity, water and sanitation facilities. There is no public education or health facilities in the slum - various NGOs provide these services. There are around 14 NGOs running 31 schools, 16 NGOs providing micro-credit loans, and 10 NGOs delivering primary health care (PHC) services. Besides, three NGOs are working to improve the environmental situation of the slum. Economic activities are centred around several *bazaars *(*jamaibazar, boubazar *etc.) scattered in different places of the slum. Majority of the dwellers earn their livelihood by wage-labour in garments and construction industries, and various self-employment activities such as pulling rickshaw, or ferrying people in boats.

### Influential people in the slum relevant to MNCH care [11]

The study tried to identify key influential people in the slum who have had a stake in the MNCH related care (n = 189) through 17 listing exercises. Though these influential people were involved with different social groups and organizations, their connection with political parties primarily enabled them to exert influence on the slum dwellers. A number of FGDs and in-depth interviews were conducted from a sample of these people to assess their awareness on MNCH issues. Findings reveal that they were quite knowledgeable about the common problems during pregnancy, labour and post-partum period, and about the problems of neonates and children. According to these people, they could contribute to *MANOSHI *activities by raising awareness of the community on MNCH care issues, advising to access care from relevant facilities, and helping with mobilization of the community for money and transport when there is an emergency.

### Social networks in the slum relevant to MNCH care [Alam et al. 2007, unpublished data]

A network analysis using UCINET software was done to identify different social networks (e.g., economic, health, education) operating in the slum. Findings reveal that slum dwellers have had strongest support network for social and economic problems but not as effective networks for health-related problems. In general, people sought advice from their relatives first, if they had anyone living in the slum or nearby locations. Among different types of problems, relatives played a major role when there was an economic hardship or disaster (25%) compared to 7% for health-related problems, 8% for delivery-related problems and 12% for social problems. Thus, researchers put more emphasis on inter-personal communication to reach slum dwellers with MANOSHI intervention messages instead of using the health-related social networks. Besides, they advised formation of women's support groups and MNCH committees for greater penetration of the community with IEC campaigns.

### MNCH service providers and facilities [12]

The study used a variety of qualitative and quantitative research techniques including listing (of all health care providers/facilities providing MNCH services), health resource mapping, semi-structured interviews, in-depth interviews, and informal group discussion with targeted respondents. The study identified seven categories of providers who were involved in providing MNCH services in the study slum: qualified allopathic doctors (MBBS) in the private sector, paramedics, unqualified allopathic practitioners (village doctors and drug shop attendants), homeopaths, community health workers of NGOs, traditional birth attendants (TBA)/*dais *and faith healers (e.g. *huzur, bhandari)*. These providers played important role in providing pregnancy-related services including services for neonatal and childhood illnesses. Though village doctors, homeopaths and faith-healers did not conduct delivery directly, they were sometimes called on by the TBAs to quicken labour by the harmful practice of injecting Oxytocin intravenously. Shopping around these providers also delayed referral to appropriate health facilities when complications arose. Other factors for delayed referral included financial constraints, problems with arranging transport, and supposed misbehaviour of the doctors/nurses in the hospitals/clinics. The providers were also interviewed for their current practices related to MNCH care.

The slum had only three EPI centres in the public sector and no private clinics. Most of the health facilities were run by various NGOs on an out-patient basis. However, there were quite a number of tertiary level facilities near the slum from where people access MNCH care. Trained doctors and midwives were available in large numbers in the public sector facilities. Barriers to access EmOC services from the referral facilities included financial incapacity, distance to facility and transport costs, behavior of the doctors and nurses, and gender norms, etc.

### Perception about pregnancy, delivery [Sharmin and Alam 2007, unpublished data]

Participatory methods such as free-listing, pile sorting and in-depth interview were done among a group of slum men and women to explore their perception about pregnancy and delivery. Findings reveal that men and women had different perspectives. While men were more concerned about the financial implications especially if complications arise, women were more concerned about the uncertainty of the birthing process and were afraid of losing their lives if they need to be hospitalized. Both men and women perceived delivery taking place at home as normal, irrespective of the duration of labour. Women were found to have no clear idea about the delivery process until they experienced it themselves. Similarly, men's knowledge on the process of delivery was poor unless they had to deal with any complication.

### Maternal and neonatal care practices [13]

Current practices related to maternal and neonatal care were elicited by in-depth interviews of the mothers with recent pregnancy outcome, and supplemented by observations of relevant events when feasible. It was found that pregnant women usually did not take any preparation for birth beforehand. They used to consider every pregnancy normal until complications arose. They did not perceive any need for antenatal care other than confirmation of pregnancy and reassurance about the condition of the foetus, especially if they have had a previous normal pregnancy. They would rely on TBAs and family members during pregnancy and labour and did not perceive any need for skilled birth attendant for normal delivery. Women used to share the information with others (beyond immediate family members) only when labour pain begins or when pain becomes unbearable. This is because there was a widespread belief that the more people heard about the impending delivery pain, the more would be delay in delivery, resulting in prolonged and difficult labour for the mother.

Women generally use to remain very active throughout their pregnancies. Observations during fieldwork found that pregnant women were busy with household chores such as cooking, cleaning, washing clothes, etc. as usual in a normal time. According to the women, until the first labour pain begins, they continued to do all household chores as usual. Food restrictions and food taboos were widely prevalent at different stages of pregnancy and labour. They strongly believed in supernatural world and maintained certain restrictions to avoid '*alga batas' *(bad air) including food restrictions.

According to them, the woman and newborn were perceived as impure (*napak*) from the blood and other fluids present at delivery and were bathed immediately to purify, and the vernix was often scrubbed off. While exclusive breastfeeding was rare, the newborn received pre-lacteal feeding such as honey, sugar water, etc. Mothers widely knew about colostrums and its benefits for the newborn babies, but this knowledge was not translated properly into practice. Applying substances such as mustard oil, coconut oil, boric or talcum powder, or earth from a clay oven, on the umbilical stump to facilitate rapid drying was common. Since contact with health care providers was limited (unless there was an emergency situation), family members and neighbours including landladies usually had much more influence over maternal and newborn lives.

### The 'birthing huts' of MANOSHI [14]

A two-part study was conducted on the newly launched birthing hut facilities in the slum areas of *MANOSHI *programme which also included the *Korail *slum. Conducted during the early inception phase of the programme, the first part attempted to capture the initial perceptions and expectations of the slum community regarding the birthing hut. Qualitative methods such as observation, in-depth interview, etc. were used. Findings reveal that the community was not sufficiently aware about the facilities and services available, and at what cost (initially the project charged nominal price for the services which was later waived). Women and family members expected 'one-stop' services for pregnancy and delivery including care for the children by qualified allopathic doctors. They were not comfortable with the idea of referral when complications arose because it was costly, and also the referral facilities were not responsive. However, those who have had accessed services at birthing hut had a mixed feeling; sometimes women did not like birthing hut because no injection (Inj. Oxytocin) was given. Initially, the CHW had grievances about remuneration and high dropout was a problem.

The second part of the study was done six months after its inception when the birthing huts were well functioning with trained staff and all amenities. By this time, the birthing huts were well embedded in the community as a safe and convenient place of delivery, especially for the poorest households. There were some gaps in information dissemination about the content and costs of the services from the birthing huts. Recipient of services were found to be quite satisfied, but demanded full-time presence of a medical doctor, steady flow of medicines, and intravenous administration of oxytocin by the birth attendants. The authors concluded by emphasizing the importance of IEC campaigns to prevent the informal providers from harmful practices.

## Discussion

The role of formative research in developing evidence-based interventions for MNCH care is now widely acknowledged [15-17]. Formative research combines different methods, both qualitative and quantitative, to elicit relevant data [18]. Different approaches to qualitative inquiry such as informal discussion with key informants, in-depth interview, FGDs, observation, etc. help in getting a holistic picture of the context for programme intervention [19,20]. This study was done to understand the context and existing resources of the intervention, and thereby develop and refine *MANOSHI *in urban slums of Dhaka, Bangladesh.

Findings reveal that though there are various MNCH services and providers available in the slums, its capacity to provide rational and quality services is questionable. Community has superficial knowledge of the MNCH care and services, but this is inadequate to facilitate the optimal survival of mothers and neonates. Due to economic hardships, the slum community mainly relies on cheap informal sector for seeking treatment, which at times may be harmful. The implications of these formative research findings to develop and refine the *MANOSHI *intervention are discussed below.

### Limitations

Formative research is done in the inception phase of the programme before interventions take shape and as such is limited by time and resources [7]. Also, different methods used have its own limitations. This may give rise to concerns regarding the validity and generalizability of the findings. However, triangulation of data from different sources partly overcame this limitation. Regarding generalizability, it can be said that the *Korail *slum was a typical one and fairly reflected the situation of the slums in other parts of Dhaka metropolis with respect to MNCH related services and practices. Later, the same process was repeated in another slum (*Kamrangir *Char) across the other part of the city to see if there was any variation, but the findings were found to be pretty similar.

### Implications of formative research findings for MANOSHI programme

Gaps identified in knowledge and practice with respect to MNCH care were used in designing IEC materials for building appropriate knowledge base. Knowing better the largely illiterate/semi-literate characteristic of the slum population, the formative research findings also helped the practitioners in taking decisions on appropriate delivery channels (e.g., using popular theatre and folk songs) for disseminating *MANOSHI *messages in a culture-sensitive way. The IEC materials and interpersonal communication tried to demystify the process of pregnancy and labour to alley anxiety of women vis-à-vis institutional delivery as noted in the formative research. Similarly, to overcome financial barrier to access facility-based skilled services as found in the formative research, mechanisms were developed to mobilize fund from the community (besides subsidy from programme) to cover costs of delivery including complications, if any.

Conscious efforts were taken by the programme to change harmful beliefs and practices identified through formative research such as intravenous infusion of oxytocin, delayed initiation of breastfeeding, or early bathing of the neonate. IEC materials were developed and used to educate mother and family on these issues including the importance of essential neonatal care for survival as well as post-natal care for reducing maternal morbidity.

As the formative research found social networks for health-related support to be poor, interpersonal communication with key influential persons were used to build up constituencies in support of *MANOSHI *activities in slums, especially in the first year. From the second year, MNCH committees were formed representing different groups in the community (50% women) whose main tasks were to raise awareness, identify and solve MNCH-related problems as and when these arise, and to improve the accountability of the service providers. Besides, mother support groups provided psychosocial help for the would-be mothers.

Given the large number of players who are involved in providing MNCH services in the slums as identified through the formative research, the programme followed an inclusive approach to take them into confidence. Improved relationship was built with service providers of other NGOs/public sector facilities (e.g., Urban Primary Health Care Project) in the slum through continued interactions in formal and informal meetings, seminars and workshops. Specific interventions were developed to sensitize the informal providers to obstetric emergencies and the need for immediate referral to institutional facilities.

Based upon findings from formative research on birthing huts, the registration fees were withdrawn to improve accessibility, and its content and form modified including attempts to address the discontents among staff which interfered with performance. Deployment of midwives reinforced trust and confidence of the community in the technical skills of the *MANOSHI *programme to offer better care for mothers and newborns. Timely physical and financial support in hospitals also helped in confidence-building and referral for complications increased. Various activities such as formation of MNCH committees and women support groups were initiated. These were instrumental in encouraging the community to use delivery centres and promote referral when complications occurred.

## Conclusions

The findings from the formative research reduced the uncertainty about the slum context with respect to MNCH care and informed *MANOSHI *programme to build up an intervention which was relevant and responsive to the felt needs of the slum population. A conceptual framework helped the researchers to stay focused while collecting data, and to triangulate information. The thematic modules developed through formative research in the initial phase of the study were used for later scaling-up of intervention in new slums in and outside Dhaka.

## Competing interests

The authors declare that they have no competing interests.

## Authors' contributions

SMA conceptualized and drafted the manuscript; he also participated in design, interpretation of data, and drafting report for the studies on mapping MNCH care resources, and the initial perceptions about the Birthing Huts. Revised and prepared the final draft of the manuscript.

AH, MAK, MKM, AA, NC, TS: principal investigators respectively of the individual studies on mapping MNCH resources, birthing huts, community groups, social networks, existing practices, perception of pregnancy and delivery.

KA, AB: helped in conceptualization of the manuscript and interpretation of data in individual studies; contributed in the critical revision of the manuscript.

All authors went through the final draft and approved it for submission.

## Pre-publication history

The pre-publication history for this paper can be accessed here:

http://www.biomedcentral.com/1471-2458/10/663/prepub
